# Obtaining Natural Sit-to-Stand Motion with a Biomimetic Controller for Powered Knee Prostheses

**DOI:** 10.1155/2017/3850351

**Published:** 2017-09-18

**Authors:** Molei Wu, Md Rejwanul Haque, Xiangrong Shen

**Affiliations:** Department of Mechanical Engineering, The University of Alabama, 286 Hardaway Hall, P.O. Box 870276, Tuscaloosa, AL 35487-0276, USA

## Abstract

Standing up from a seated position is a common activity in people's daily life. However, for transfemoral (i.e., above-knee) amputees fitted with traditional passive prostheses, the sit-to-stand (STS) transition is highly challenging, due to the inability of the prosthetic joints in generating torque and power output. In this paper, the authors present a new STS control approach for powered lower limb prostheses, which is able to regulate the power delivery of the prosthetic knee joint to obtain natural STS motion similar to that displayed by healthy subjects. Mimicking the dynamic behavior of the knee in the STS, a unified control structure provides the desired control actions by combining an impedance function with a time-based ramp-up function. The former provides the gradual energy release behavior desired in the rising phase, while the latter provides the gradual energy injection behavior desired in the loading phase. This simple and intuitive control structure automates the transition between the two phases, eliminating the need for explicit phase transition and facilitating the implementation in powered prostheses. Human testing results demonstrated that this new control approach is able to generate a natural standing-up motion, which is well coordinated with the user's healthy-side motion in the STS process.

## 1. Introduction

Standing from a seated position is a common yet dynamically challenging task in people's daily life. Due to the vertical ascent of the body's center of gravity, sit-to-stand (STS) transition requires high torque in the knee, far exceeding the joint torque in walking. Various biomechanical studies reported knee peak torques in STS as high as 2.2 Nm/kg (e.g., [1]), while the typical peak torque in natural walking is only 0.615 Nm/kg [2] (both body weight normalized). As a result, STS transition poses a major barrier to the mobility of individuals with lower limb motor impairments, including the transfemoral (TF) amputees (i.e., individuals suffering from above-knee amputations). A related study showed that TF amputees suffer from much higher asymmetry in ground reaction force and knee moments in the sit-to-stand motion, compared with no-amputee healthy individuals [3]. According to the results of this study, the healthy individuals' ground reaction force and knee moment production were less than 7% asymmetric, while the amputees' asymmetry for ground reaction force was 53~69%, and the asymmetry for knee moments was 110~124%. Note that although a powered TF prosthesis (Power Knee) was used in this study, it generated resistance in the STS and thus produced similar results as the passive devices in the study (C-Leg and Mauch SNS). The inability of existing prostheses in generating enough knee torque and regulating the torque delivery in the STS seriously affects the mobility of the large population of TF amputees in their daily life.

Motivated by this significant performance deficiency of traditional passive TF prostheses, researchers have expended substantial efforts in developing powered devices. The pioneering work in this area was conducted by Flowers and Mann, which uses a hydraulic actuator to actuate the knee joint [4]. However, multiple drawbacks with hydraulics, such as leakage and lack of a compact supply, make it less attractive for prosthetic applications. Currently, most powered transfemoral prostheses are actuated with electric motors [5–10], for example, Sup et al. developed a powered knee and ankle prosthesis with both joints powered with a DC motor—ball screw assemblies [8]; Martinez-Villalpando and Herr developed a powered knee prosthesis with two series-elastic actuators positioned in parallel in an agonist-antagonist arrangement [9]; and Hoover et al. developed a myoelectric transfemoral prosthesis, in which the powered knee is controlled with an EMG-based motion controller [10]. Additionally, Ossur, a leading orthopedics company, manufactured the aforementioned Power Knee, the first commercially available powered transfemoral prosthesis. According to the available technical information, the Power Knee is also actuated with an electric motor. In addition to these motor-powered devices, pneumatically actuated prostheses have also been developed by researchers including the authors' group, for example, the prototypes powered with pneumatic cylinders [11] and muscle actuators [12].

The powered prostheses mentioned above are able to actively generate joint torque and power for dynamically challenging tasks such as STS transition. Making full use of such capability, however, requires an effective and reliable controller to regulate the joint power delivery in motion. Currently, a walking controller for powered prostheses has been well established. Typical approaches include echo control, which controls the prosthetic joint to track the recorded sound-side motion with a half-cycle delay [13], and finite-state impedance control, which implements an artificial impedance within each phase of the gait cycle [11]. Electromyography has also been attempted in obtaining the user's motion intent and generating the corresponding motion command [14]. STS control, however, is much less investigated. The Center for Intelligent Mechatronics at Vanderbilt University developed a multimode controller for powered knee and ankle prostheses, in which STS is incorporated as a transitional motion between sitting and standing states [15, 16]. However, no details were provided on the rationale of the controller structure or the determination of the control parameters.

In the research presented in this paper, the authors developed a new control approach to regulate the power and torque delivery in the STS process. As the basis of this approach, an analysis of the biomechanical behavior of the knee in the STS was conducted, providing the inspiration for the proposed controller structure. Subsequently, curve fitting was conducted to evaluate the validity of the new controller structure, utilizing the existing biomechanical data of the STS motion. This new approach was implemented in a powered knee prosthesis developed in the authors' lab, generating qualitative and quantitative results to evaluate its effectiveness.

## 2. Knee Biomechanical Behavior-Inspired STS Controller

Biomechanics in STS is a heavily investigated topic with a large body of data generated from numerous studies. Ideally, an STS controller should replicate the biomechanical behavior of the knee in this process, providing the prosthesis user a natural control experience. However, exactly replicating the kinetic and kinematic trajectories of the biological knee is unfeasible. Human locomotion is a highly interactive process, in which the human lower limbs interact with the rest of the human body and the environment to obtain coordinated motion. Enforcing the kinetic/kinematic trajectories in the prosthetic knee precludes such interaction, resulting in a poor experience for the prosthesis users. Results of the biomechanical data, on the other hand, provide insight to the dynamic behavior of the knee and thus can be used as the inspiration for the prosthesis controller.

**Figure 1:**
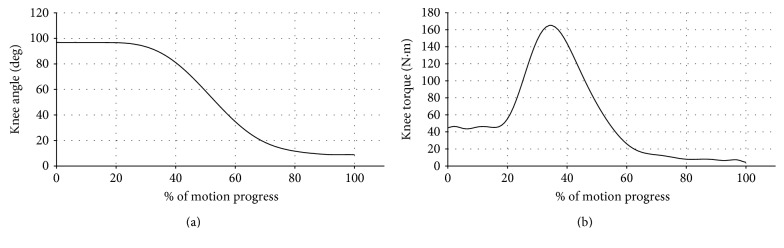
Knee position (a) and torque (b) trajectories in the STS motion (plotted for a 75 kg person with the data from [1]).

Unlike cyclical motion modes such as walking, STS is a typical transitional motion with clearly defined start (seated position) and end (standing position). The typical joint position and torque trajectories are shown in Figure 1 (data from [1]). The entire process can be divided into two phases with distinct dynamic characteristics, separated by the instant of seat-off (SO):

1. Loading phase (from start to SO): with the body weight shifted from the seat to the lower limb, the knee torque increases rapidly to support the body weight and initiate the upward motion. In this phase, the knee position remains almost constant until the final portion of the phase, and the torque increases at a nearly constant rate after the initial dormant period.
2. Rising phase (from SO to end): after reaching the maximum value, the knee torque reduces with the joint extension and settles at a steady-state value after the standing position is reached.

Such segmentation of the STS motion can be clearly seen in Figure 1. For a powered knee prosthesis to generate natural motion in this process, the controller should follow the same strategy, generating a knee torque that changes in a way similar to the biological joint torque trajectory. To facilitate the implementation in powered prostheses, the STS controller structure should be adequately simplified while retaining the essence of human biological control. Furthermore, considering the significant intersubject variation among prosthesis users, subject-specific tuning is an indispensable step in fitting a powered prosthesis. Ideally, the number of control parameters should be minimized, and all parameters should have clearly defined physical meanings to make the tuning process intuitive and easy to understand. Based on the multiple requirements above, the authors propose a control structure consisting of a time-based ramp-up function for gradual loading of the knee combined with an impedance function for automatic adjustment of knee torque according to the motion progress:

(1) $$\begin{array}{c} \tau =R\left(t\right)\cdot \tau_{imp}\left(\theta ,\dot{\theta }\right). \end{array}$$

In this equation, the impedance function *τ*_imp_ is defined as

(2) $$\begin{array}{c} \tau_{imp}=K\left(\theta -\theta_{e}\right)+B\dot{\theta }, \end{array}$$

where *θ* is the joint position (measured from the knee-straight position), $\dot{\theta }$ is the joint angular velocity, *K* is the stiffness of the virtual spring, *θ*_e_ is the equilibrium point of the virtual spring, and *B* is the damping value of the virtual damper. The ramp-up function *R*(*t*) is defined as

(3) $$\begin{array}{c} R\left(t\right)=\left\{\begin{array}{cc} \frac{t-t_{0}}{T} & when\;t_{0}\leq t\leq t_{0}+T\\ 1 & when\;t>t_{0}+T, \end{array}\right. \end{array}$$

where *t* is the current time point, *t*_0_ is the starting time point of the ramp-up period, and *T* is the length of the ramp-up period.

The impedance function, as the major part of the controller, simulates the dynamics of a mechanical spring combined with a viscous damper. A mechanical spring is energetically conservative, while a viscous damper is dissipative. As such, the simulated spring-damper combination is purely passive, guaranteeing the stability in the control process. The passivity, on the other hand, dictates that all the required artificial mechanical energy (in the form of artificial spring deflection) is to be introduced at the onset of STS motion, such that sufficient power output can be provided while lifting the user in the upward motion. Consequently, the torque output of the spring-damper combination immediately reaches the maximum at the motion onset, as opposed to the gradual increase as shown in the biomechanical data.

The time-based ramp-up function is then introduced to address this problem. As (3) shows, the value of the function increases linearly from 0 to 1 within the ramp-up period and stays at 1 afterwards. As such, it only takes effect in the ramp-up period, providing the gradual energy injection required in the loading phase. It is worth mentioning that the use of the ramp-up function eliminates the need for explicit phase transition from loading to rising as a result of the limited effective period, which significantly simplifies the implementation of controller. Note that other functions may also be used to generate the gradual loading effect in the load phase. For example, a sigmoid function (*y* = 1/(1 + *e*^−*x*^)) also monotonically increases from 0 to 1, with the additional advantage of having a continuous derivative. However, such function usually comes with higher computation load than the ramp-up function in the real-time implementation. Furthermore, the ramp-up function has a tunable parameter *T* that has a clearly defined physical meaning (the duration of the ramp-up period). As such, it is easier to tune the speed of loading to fit each individual user when the ramp-up function is used.

**Figure 2:**
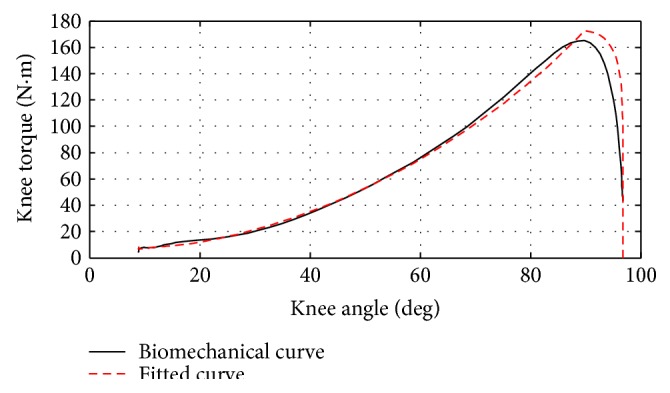
Comparison of the fitted knee torque curve versus the knee torque curve in the biomechanical data for the STS motion [1].

To validate the controller structure shown by (1), (2), and (3), curve fitting was conducted based on the biomechanical data of knee position/velocity and torque in the STS for a 75 kg subject [1]. Matlab Curve Fitting Toolbox was utilized to obtain the optimal set of values for *K*, *θ*_e_, *B*, and *T* with the least error from the biomechanical data.  Figure 2 shows the comparison of the fitted knee torque curve versus the knee torque trajectory plotted from biomechanical data. The close match between the two curves indicates that the proposed controller structure is able to replicate the dynamic behavior of the biological knee joint in the STS motion with very small error.

Finally, to initiate the control action, the axial load in the prosthesis combined with the knee joint angle serves as the indicator for the user's readiness for the STS motion. When the user prepares for standing up, he/she first bends the knees by a large angle (usually greater than 90°) such that the feet can be directly underneath the body's center of mass. Subsequently, the weight is gradually shifted to the feet, increasing the axial load born by the prosthesis. Based on such biomechanical process, the trigger condition is set as the prosthesis axial load greater than a certain threshold *F*_T_, and the prosthesis knee angle also greater than a certain threshold *θ*_T_. This simple yet effective triggering condition can be easily implemented by using the embedded sensors in the prosthesis and provides an intuitive and reliable way to initiate the STS motion. If the axial load information is not available, the inclination of the upper body may also serve as a triggering condition, as a user usually leans forward significantly when attempting to stand up. Alternatively, direct human input (e.g., through voice command or a switch) may also be used for this purpose.

## 3. Human Testing Results

**Figure 3:**
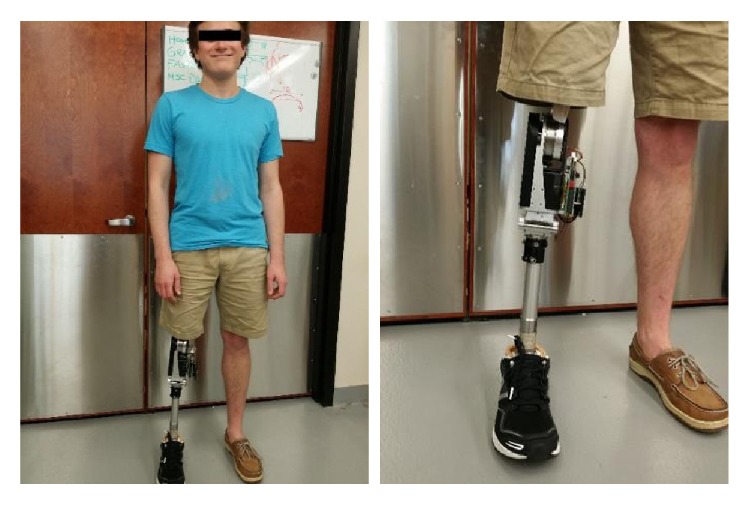
The test subject fitted with the powered knee prosthesis.

To demonstrate the effectiveness of the STS controller, the authors conducted a set of human subject experiments at the Human-Centered BioRobotics (HUB-Robotics) Laboratory at The University of Alabama. The human subject participated in the testing was a 22-year-old male unilateral amputee, 178 cm in height and weighed 57 kg. He was fitted with a powered knee prosthesis prototype developed at HUB-Robotics Lab, namely Alabama Powered Prosthetic Limb-Knee (APPL-K), shown in Figure 3.

The version of the powered knee prosthesis used in this study, APPL-K-E1, was powered by an 8-pole brushless DC motor with 70 W power rating (EC 45 flat, Maxon Motor, Sachseln, Switzerland). For short-term operation, this motor is able to generate a peak torque of 0.2 Nm and a maximum rotation speed of 10,000 rpm. A two-stage transmission of 150 : 1 gear ratio is used, combining a timing belt drive as the first stage and a harmonic drive gearhead as the second stage. Note that, in the design of the device, reducing the weight and simplifying the system structure was given higher priority than generating higher torque output, and the actuation torque is less than the peak value in the biomechanical data. This issue, however, did not affect the performance of the prosthesis in the STS, as indicated by the experimental results below.

For the implementation of the STS controller, the prosthesis is instrumented with various control components for computing, sensing and regulation of power delivery. The joint position is measured with a rotary magnetic encoder, and the position signal is digitally differentiated to obtain the joint angular velocity information. A custom load cell developed by the Center for Intelligent Mechatronics at Vanderbilt University [17] is mounted between the prosthesis and the standard pyramid connector to measure the axial force in the prosthesis. The power output of the DC motor is regulated with a PWM servo drive (AZBDC20A8, Advanced Motion Controls, Camarillo, CA, USA), which controls the motor current as a function of the PWM duty cycle. The controller is implemented on a microcontroller (Microstick II, Microchip Technology Inc., Chandler, AZ, USA), which communicates with a host desktop computer to record and display experimental results for controller tuning and data analysis.

Due to the limitation of available equipment, the data collected in the testing were all based on the sensors embedded in the prosthesis, primarily the joint angle and torque trajectories. After being fitted with the powered prosthesis, the subject repeated the STS motion to identify the best set of control parameters through his feedback and recorded experimented data. The stiffness of the artificial spring in the controller *K* was increased gradually, providing increasing extensional torque to assist the user to stand up. The final value, with which the user is most comfortable with, generates a peak torque of 25 Nm, much less than that in the biomechanical data. The primary reason, presumably, is that the subject is used to the lack of power supply in his daily-use passive prosthesis, and thus the comfortable level of power supply in his prosthetic joint has been reduced significantly compared with that in a healthy biological joint. This observation, to some extent, validates the original decision of prioritizing low weight over high torque in the prosthesis design.

The damping of the artificial viscous damper was also tuned. With the function of controlling the speed of standing up, the damper reduces the extensional torque or even generates a flexional torque if the extension of the knee becomes too fast. The damping value was also adjusted primarily according to the feedback of the test subject. The finalized controller parameters are shown in Table 1.

**Figure 4:**
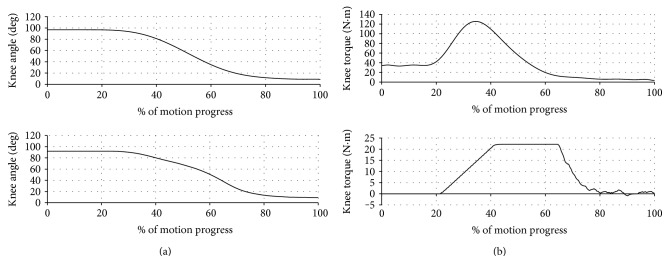
Comparison of the typical trajectories of the prosthetic joint versus the biomechanical trajectories in [1]: (a) angle trajectory comparison, with the biomechanical trajectory above the experimental trajectory; (b) torque trajectory comparison, with the biomechanical trajectory (for a 57 kg person) above the experimental trajectory.

**Figure 5:**
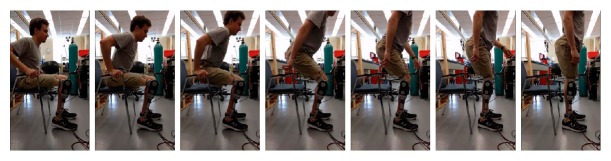
A sequence of snapshots of the STS motion.

The typical trajectories of the experimentally measured prosthetic joint position and torque are shown in Figure 4, and a sequence of snapshots of the STS process is shown in Figure 5. The data window started when the triggering condition was met. As can be seen in Figure 4, the joint position stayed almost as a constant until the rising phase started, and the whole trajectory shows smooth and controlled motion throughout the process. Compared with the biomechanical data shown in Figure 1, the contour of the experimentally measured prosthetic position trajectory is highly similar. For the joint torque trajectory, the dynamics of the loading and rising phases can be clearly identified and distinguished, while the flat peak in the middle of the cycle is a result of the torque saturation (i.e., reaching the maximum torque as dictated by the prosthetic actuator). The overall contour is also similar to that of the biomechanical curve in Figure 1. Matching the observations from these figures, the subject also stated a natural control experience in which the prosthesis motion coordinates with the sound-side leg motion well, and the extensional torque in the prosthesis enabled him to stand up with less effort. Such quantitative and qualitative results fully demonstrated the effectiveness of the proposed controller, which provides the prosthesis user a significantly improved experience over the traditional passive prosthesis.

## 4. Conclusions

In this paper, the authors present a new control approach for powered knee prostheses in the STS motion. The objective was to develop an STS controller that regulates the extensional torque in powered knee prostheses to obtain smooth standing-up motion. As the basis of the controller development, the biomechanical data from prior STS studies were analyzed. The dynamics in the loading and rising phases are vastly different. However, a unique control structure was created, which combines the impedance function with a time-based ramp-up function. The impedance function was introduced to provide the gradual energy release in the rising phase, while the ramp-up function was included to mimic the gradual energy injection behavior in the loading phase. The use of such unified control structure simplifies the controller implementation while maintaining the unique biomechanical characteristics of each motion phase. This new STS controller was implemented on a powered knee prosthesis developed in the authors' lab, and human testing results demonstrated the effectiveness of this approach in generating smooth standing-up motion according to the user's will.

## Figures and Tables

**Table 1:** The parameters of the STS controller.

*T* (s)	*K* (Nm/deg)	*B* (Nm·s/deg)	*θ*_0_ (deg)
0.47	1.1	0.4	8
